# The COVID-19 Infodemic Through Facebook: Comparison of Content and the Accuracy of Breastfeeding Information

**DOI:** 10.7759/cureus.23734

**Published:** 2022-04-01

**Authors:** Nurul Husna Mohd Shukri, Nursaleha Mohd Sobri, Siti Rohkmah Mohd Shukri

**Affiliations:** 1 Department of Nutrition, Faculty of Medicine and Health Sciences, Universiti Putra Malaysia, Serdang, MYS; 2 Institute for Social Science Studies, Universiti Putra Malaysia, Serdang, MYS; 3 School of Information Technology, Monash University Malaysia, Subang Jaya, MYS

**Keywords:** seeking information, nursing, parenting, health information, infant nutrition, maternal perception, breastfeeding education, infant feeding, social media, lactation

## Abstract

Background

The coronavirus disease (COVID-19) pandemic has resulted in a significant increase in the number of people seeking online support and information, particularly on social media. Nevertheless, the nature and trend of internet information, as well as its accuracy, are questionable. This study aimed to assess and compare the content, type/form, and degree of accuracy of breastfeeding information on Facebook before and during the COVID-19 pandemic (n = 288/phase).

Methodology

The data were gathered from Malaysian public and group/page Facebook posts (n = 456). Keyword searches were conducted using Malay and English breastfeeding terms. The dataset was screened and entered into a structured codebook. The Delphi approach was used to assess the accuracy of posts’ content performed by breastfeeding experts.

Results

Sharing personal experience (53.2%) was the most common topic in breastfeeding-related posts, followed by seeking questions (39.3%) and knowledge (8.0%). Sharing personal stories and knowledge posts were higher during COVID-19 than before (p = 0.001), although the seeking questions category was higher before the pandemic (p = 0.001). Most information posted was in text form (94.5%). About half of the posts (46.5%) were misleading, while (43.7%) were accurate. There was a significant difference in the accuracy of online posts before and during the COVID-19 pandemic (p = 0.001).

Conclusions

Compared to the pre-pandemic phase, forms/types of information on Facebook remained consistent, whereas the breastfeeding information content and its degree of accuracy differed during the pandemic.We need to explore other aspects of breastfeeding online content as well as its engagement, especially during a pandemic. Knowing the infant feeding-related topics that have been discussed and questioned on social media, as well as the accuracy of the data, allows policymakers and scientific communities to plan strategies for spreading credible breastfeeding information online. This includes creating interactive online media types of visual guidelines, web resources, and breastfeeding apps.

## Introduction

Breastfeeding is the gold standard for newborn nutrition in the first few months of life. It has shown benefits in reducing infections in infants and lowering the risk of non-communicable diseases, such as diabetes and obesity, during adulthood [1]. Moreover, children who were exclusively breastfed have shown better cognitive and intellectual development, whereas their mothers were more protected from breast and ovarian cancer [1]. In Malaysia specifically, the National Health and Morbidity Survey (2016) reported that less than half of the infants (47.1%) were exclusively breastfed in the first six months of life [2]. Due to the importance of breastfeeding, government and non-governmental organizations (NGOs) have made efforts to enhance breastfeeding rates to meet the aim of achieving 70% exclusive breastfeeding rates in Malaysia by 2025.

On March 11, 2020, the World Health Organization proclaimed the coronavirus disease 2019 (COVID-19) outbreak as a pandemic. How severe acute respiratory syndrome coronavirus 2 (SARS-CoV-2) could be transmitted during breastfeeding activities was worrisome at the time [3]. Hence, some countries started separating mothers from their newborns at birth during this period, which has been a major concern for infant health [4]. In the UK, seven out of ten mothers lack face-to-face support during the pandemic [5]. In addition, mothers in the UK have also expressed their intentions of ceasing breastfeeding due to the pandemic. Overall, there was a reduction in essential support from families and friends in maintaining the breastfeeding journey among mothers [5]. Hence, social media has increasingly been used as a sharing medium to exchange knowledge and experience among breastfeeding mother communities. Healthcare experts are also now tuning into social media for discussion on appropriate methods of breastfeeding practices for parents.

Social media in Southeast Asia has reached around 482.73 million individuals [6]. In Malaysia alone, the Malaysian Communication and Multimedia Commission [7] reported that 81% of Malaysians are active social media users, mainly on Facebook, Twitter, Instagram, and YouTube. Roselina et al. (2021) reported that a high majority (78.3%) of the study population chose social media to seek information regarding COVID-19 [8]. In particular, 76% of users relied on social media such as Facebook and Twitter for health information during COVID-19, in addition to gathering information from websites [9]. Even most face-to-face support groups have switched to online or social media [5]. According to Statista (2020), Facebook has a 70% penetration rate in Malaysia [6]. In healthcare, patients’ primary motivations for utilizing Facebook are social support, advice exchange, and knowledge expansion [10]. Particularly, the use of Facebook groups has proven to be an effective communication and information-gathering medium for many areas, such as depression and breast cancer [11]. Moreover, Kallem et al. (2018) found that Facebook groups are mostly used by mothers to ask questions on common topics regarding infant health, such as solid food introduction, teething, and breastfeeding [12]. Therefore, we are interested in analyzing how mothers communicate using Facebook groups in the breastfeeding area.

However, the lack of trustworthiness of health information on social media needs to be discussed [1]. There is an abundance of false materials on social media with the potential for misinformation. For example, in Iran, over 700 people died after consuming alcohol (toxic methanol), thinking it could cure coronavirus [13]. Atehortua and Patino (2021) found that more than half of the health-related information regarding COVID-19 was fake (60.4%) [14]. This includes false SARS-CoV-2 information, hoax or fake pandemic and anti-vaccine messaging, fake prevention and treatment, and home remedy ideas. The most popular platform for spreading fake news is Facebook (38.0%), followed by Twitter (16.7%), WhatsApp (13.7%), and YouTube (5.0%) [15]. How reliable is the information that is being spread across social media platforms? This has motivated us to capture content information exchange on Facebook within the scope of breastfeeding information.

Therefore, our study aimed to examine the content of type or form of breastfeeding information on Facebook. We intended to compare the data dissemination before and during the COVID-19 pandemic. This includes investigating the degree of accuracy of breastfeeding information shared on Facebook. We hope our findings can benefit communities, as well as policymakers and scientists in the area.

## Materials and methods

Study design

A total of 456 data points have been included in this study. The sample size was calculated using the reference of the hypothesis testing of two population proportion studies. The data were collected in two phases, namely, pre-pandemic and during the COVID-19 pandemic. The first phase was six months before the pandemic (from September 01, 2019, to February 29, 2020), whereas the second phase was during the eight months of the pandemic (from March 01, 2020, to October 31, 2020). The month of March was chosen due to the start of the Malaysian Government Movement Control Order (MCO), or the partial lockdown, going into effect. The following eight months comprised all three significant waves of COVID-19 cases in Malaysia affected by the Enhanced Movement Control Order (EMCO) and Conditional Movement Control Order (CMCO) in many places in Malaysia such as Sabah, Kedah, and Selangor. Hence, six months of the pre-pandemic phase were compared to the eight months of the pandemic phase.

Research procedures

Social media, specifically Facebook, was chosen as the data collection platform in this study due to its high penetration rate (70%) and because it is considered the most popular social media platform in Malaysia [6]. Moreover, Facebook is estimated to have around 24 million members in 2023, according to Statista (2020) [6]. In this study, Facebook posts were collected purposively by keyword insertion using Google Trends and Mesh Browser terms relating to breastfeeding. The generated keywords used were in English and Malay (e.g., English: breastfeeding, breastfeed, breast milk; Malay: *menyusu, susu ibu, penyusuan susu ibu*). The keywords were inserted in public posts and groups on Facebook, such as groups related to parenting, pregnancy, breastfeeding, and newborn care. Public groups were chosen based on the criteria that most users were Malaysian and the groups were online shopping for breastfeeding or newborn group, but rather online social support groups on Facebook. The data collected were screened according to the inclusion and exclusion criteria of the study. Data that met the inclusion criteria were then keyed in and organized into a codebook.

Data management

The codebook used in this study was adopted from previous studies [11,15-17]. It consisted of four main sections, starting with information about the online Facebook posts’ details and topics, followed by the breastfeeding information content, forms/types of breastfeeding information posts, and, finally, the degree of accuracy of breastfeeding information. After the data were entered into the codebook, the degree of accuracy of the data was analyzed. The degree of accuracy of breastfeeding information was divided into four categories: (i) correct information, (ii) misleading information, (iii) false information, and (iv) no information. The Delphi approach was used to re-check the accuracy of post content by experts after being analyzed according to established policies and guidelines. The examples of the guidelines were the Breastfeeding Manual and Guidelines developed by the Malaysia Ministry of Health and the breastfeeding guides and information from the World Health Organization and United Nations Children’s Fund. The experts involved in the Delphi method were a nutritionist and a public health specialist, both of whom had completed their doctorate degrees in the infant nutrition field. Both experts are lactation counselors and have actively conducted research and public health education on breastfeeding in Malaysia.

Data analysis

SPSS version 26 (IBM Corp., Armonk, NY, USA) was used to analyze the data. Univariate analysis was used to analyze descriptive data such as frequency and percentage. The study’s hypotheses were tested using bivariate data analysis. Next, the chi-square test was performed to determine the differences in categorical variables between the two time periods (before and during the COVID-19 pandemic). If certain assumptions were not met, Fisher’s exact test was performed. The threshold for statistical significance was set at p < 0.05.

## Results

Breastfeeding information posts

As shown in Table 1, combining all posts pre-pandemic and during the pandemic, sharing personal experiences accounted for most of the content (53.2%) on Facebook, followed by 32.0% of posts seeking questions/advice/support. Research updates and policy/interim guaranty (0.6%) were the least popular breastfeeding information topics. For topics on breastfeeding content, breastfeeding management was the most popular (36.9%), followed by 14.5% for breastfeeding and health. As shown in Table 1, text was the most common form or type of breastfeeding information (94.5%).

**Table 1:** Comparison of content and forms/type of breastfeeding information between pre-pandemic and the COVID-19 pandemic (n = 456). COVID-19: coronavirus disease 2019

	Before COVID-19, N (%)	During COVID-19, N (%)	Total data, N (%)	χ^2^	P-value
Breastfeeding information content			286 (53.2)		
Sharing personal stories	126 (44.1)	160 (55.9)	172 (32.0)	10.21	0.001**
Seeking answers/advice/support	103 (59.9)	69 (40.1)	43 (8.0)	10.17	0.001**
Knowledge	20 (46.5)	23 (53.5)	26 (4.8)	0.10	0.749
Advertisement/Claimed post	23 (88.5)	3 (11.5)	5 (0.9)	14.72	0.001**
Others	-	5 (100.0)	3 (0.6)		0.061
Research update	2 (66.7)	1 (33.3)	3 (0.6)		1.000
Policy/Interim guideline	-	3 (100.0)			0.248
Topics of the posts					
Breastfeeding management	52 (58.4)	37 (41.6)	89 (36.9)	2.74	0.098
Breastfeeding and health	24 (68.6)	11 (31.4)	35 (14.5)	4.46	0.035**
Express breast milk	16 (57.1)	12 (42.9)	28 (11.6)	0.34	0.558
Breastmilk sharing	10 (40.0)	15 (60.0)	25 (10.4)	0.68	0.411
Low supply	18 (81.8)	4 (18.2)	22 (9.1)	8.07	0.004**
Breastfeeding and medication/drugs/alcohol	6 (54.5)	5 (45.5)	11 (4.6)	0.00	1.000
Breastfeeding and clinical issues	3 (33.3)	6 (66.7)	9 (3.7)		0.503
Breastfeeding and work	5 (55.6)	4 (44.4)	9 (3.7)		1.000
Related complementary feeding	2 (25.0)	6 (75.0)	8 (3.3)		0.285
Weaning/Stopping breastfeeding	2 (40.0)	3 (60.0)	5 (2.1)		1.000
Type/Forms of breastfeeding information				1.28	0.716
Text	218 (50.6)	213 (49.4)	431 (94.5)		
Infographic	6 (42.9)	8 (57.1)	14 (3.1)		
Video	3 (37.5)	5 (62.5)	8 (1.8)		
Photo	1 (33.3)	2 (66.7)	3 (0.7)		

Accuracy of breastfeeding-related information posts

Only 71 (15.6%) of the posts were judged for their accuracy (Table 2). Sharing stories and asking questions/advice are examples of information that was not relevant to be assessed for accuracy. The majority of the accuracy of breastfeeding information was misleading information (46.5%), correct information (43.7%), and mixed information (9.9%). For each accuracy level, the accuracy scale was further examined. Overall, 84.8% of the information was misleading on the misleading scale. Strongly correct accounted for 41.9% of the correct information. Both somehow-correct and mixed-information had the highest percentages (42.9%).

**Table 2:** Comparison of the degree of the accuracy of breastfeeding information between pre-pandemic and the COVID-19 pandemic. COVID-19: coronavirus disease 2019

	Before COVID-19, N (%)	During COVID-19, N (%)	Total data, N (%)	χ^2^	P-value
Degree of the accuracy of breastfeeding information	42 (59.2)	29 (40.8)	71 (100)	14.42	0.001**
Misleading information	27 (81.8)	6 (18.2)	33 (46.5)		
Correct information	11 (35.5)	20 (64.5)	31 (43.7)		
Mixed information	4 (57.1)	3 (42.9)	7 (9.9)		

Comparison of content, forms/types, and accuracy of breastfeeding information before and during the COVID-19 pandemic

There were significant differences in the total number of posts related to sharing personal stories (χ^2^ = 10.21, p = 0.001), seeking questions/advice/support posts (χ^2 ^= 10.17, p = 0.001), and advertisement/claimed posts (χ^2^ = 14.72, p = 0.001) before and during COVID-19, as indicated in Table 1. In terms of posts, there was a significant difference between before and during COVID-19 in the total of breastfeeding and health (χ^2 ^= 4.46, p = 0.035) and low supply (χ^2 ^= 8.07, p = 0.004). The type/forms of breastfeeding information used by the user before and during COVID-19 were not significantly different (χ^2^ = 1.28, p = 0.716). The degree of accuracy of breastfeeding information before and during COVID-19 differed significantly in the number of total posts (χ^2^ = 14.42, p = 0.001).

## Discussion

This study compared the content of breastfeeding information sought before and during the pandemic in Malaysia. This is the first social media content analysis conducted in Malaysia on infant feeding comprising a large sample size of 456 posts. Overall, our study shows that most breastfeeding content on social media was sharing personal stories, follow-up with seeking answers/advice/support posts, and knowledge-informative posts. In addition, the most frequent topic under the breastfeeding content posted by the user was breastfeeding management, with the text being the most used form/type of information. In terms of post accuracy, almost half of the data were considered misleading, and the other half was correct, with minor mixed information. On comparing the two periods, before and during the COVID-19 pandemic, there were significant differences in the total number of posts related to sharing personal stories, seeking answers/advice/support posts, and advertisement/claimed posts. The user’s type/forms of breastfeeding information posts were not significantly different. The degree of accuracy of breastfeeding information differed significantly in the number of total posts.

Breastfeeding information content

Posts on sharing personal stories focused primarily on their infant’s development, breastfeeding progress, and mothers’ breastfeeding experiences. Consistently, according to Chalklen and Anderson (2017), more than half of the mothers (60.6%) posted about their children’s development or progress for sharing purposes [18].

Uncertainty or asking for an opinion on problems their babies face during the breastfeeding journey was included under the online post category of seeking questions, advice, or support. Users also seek assistance from online peers or other mothers in enduring their breastfeeding journey. More than half of the mothers (6.0%) sought help maintaining breastfeeding, protecting their infants by breastfeeding, and boosting their milk supply knowledge. A previous study has shown that seven out of ten parents believe Facebook assisted them in resolving parenting issues and made them feel less alone in struggling to be better parents [18]. According to Kallem et al. (2018), mothers primarily used Facebook groups to ask questions [12]. Most questions were about newborn growth and health, with many focusing on feeding (breastfeeding and solid feeding) and teething issues.

In the knowledge context, breastfeeding information was included. Government and health professionals were the most common sources of information. However, other users also contributed. This could be due to the growing concern of users looking for health-related information. A recent study reported that one of the main reasons mothers use the internet is to find parenting and health information regarding their infant [19]. This study did not include advertisements for breastfeeding products such as breast pumps or milk storage, except for milk boosters. Milk boosters should be substituted with education online to counteract the false information about milk boosters. In Indonesia, public healthcare provided a breastfeeding booster package that included information about the causes of decreased breast milk production, oxytocin massage, and strategies to increase breast milk production, all of which have been shown to improve breastfeeding knowledge among mothers [20].

Breastfeeding management accounted for most of the data collected in this study, followed by breastfeeding and health and breastmilk. This finding is comparable to Bridges et al. (2018) who found that breastfeeding management and breastfeeding and health were the most prevalent searches [16]. Another recent study reported that feeding challenges, breast milk supply issues, and nursing feeding schedules were the most common topics for mothers looking for information online [21]. Regarding infant topics, mothers tend to seek information about infant complementary feeding, nursing, and teething during the postpartum period [22]; hence, these topics should be addressed more thoroughly when educating mothers during postpartum health visits. The two primary types of breastfeeding content are sharing personal stories and seeking questions, mostly posted in text forms. This finding was consistent with a study on public diabetes Facebook groups, which reported that text was the most common form of posts on Facebook walls, followed by other types of media such as photos and videos [23]. In contrast, in a public health communication study among Australian users on Facebook, photo posts were the most popular post type, followed by link-sharing and video. Furthermore, video posts received the highest engagement and attention (e.g., likes, shares, and comments) compared to other types of posts on Facebook [24]. Consistently, texts and links received less engagement than photographs and videos, as shown by fewer likes and shares. This indicates that although the text has frequently been posted on Facebook walls, users’ engagement could be higher in other types of posts, especially videos and photos on Facebook. As visuals arouse greater human curiosity, they may be the main reason why advertising employs more visuals. For example, marketing visual media on television is more expensive than text-form advertisements in newspapers. Hence, posting visual media types on social media could be used more frequently to raise public health awareness, especially during the pandemic.

Degree accuracy of breastfeeding-related information

To improve the reliability of evaluating the degree of accuracy of breastfeeding information, the Delphi technique incorporating expert verification was applied in this study. The present study found that milk boosters-related posts and advertisements/claimed posts mostly comprised misleading information, whereas correct information came from a health professional and a health organization. Meanwhile, mixed information consisted of a combination of correct and false or misleading information or false and misleading information in a post. A study on drug misinformation on social media discovered that most drug information messages were potentially misleading, implying that the information was stated without enough evidence, which led to false claims [15]. For example, as indicated in the study, potentially misleading claims about the drug were spread on WhatsApp regarding its exaggerated negative effects and overstated clinical significance and efficacy. Hence, there is potentially misleading digital information regarding health claims or information on various social media platforms.

Comparison of content, forms/types, and accuracy of breastfeeding information before and during the COVID-19 pandemic

The number of posts seeking answers, support or advice, advertisements, and research updates before the COVID-19 pandemic was significantly higher than during the COVID-19 pandemic. On the other hand, posts for sharing personal stories and knowledge during the pandemic were significantly higher than in pre-pandemic. People may choose to share their experiences or stories and seek nursing knowledge on Facebook posts because they cannot meet face-to-face due to restrictions or lockdown during the pandemic. More than half of the mothers (61.0%) were unable to obtain face-to-face health care, and half of the mothers were afraid to visit their doctor for fear of being exposed to COVID-19, as reported in a study in Surabaya, Indonesia [25]. This has increased the number of people looking for health information on the internet. The study in Indonesia also found that 78.3% of respondents prefer to get information on COVID-19 through social media [8]. Individuals are more concerned about the COVID-19 problem than the effect of COVID-19 on breastfeeding. Only 10.0% of mothers were concerned about the link between SARS-CoV-2 and the safety of nursing [25]. Instead, mothers were more concerned about their lack of understanding of how to care for their newborns and securely breastfeed during the pandemic [26]. Hence, posts sharing personal stories appear to be the highest compared to other breastfeeding content, as well as higher frequency during the COVID-19 pandemic.

Facebook posts on breastfeeding and management, breastfeeding and health, expressed breast milk, low supply, breastfeeding, medication/drug/alcohol, and breastfeeding and work were higher before COVID-19 than during COVID-19. Breastmilk sharing, breastfeeding, and clinical issues, as well as related complementary feeding and weaning/stopping breastfeeding posts, were greater during COVID-19 than they were before COVID-19. Because of the pandemic, mothers tend to seek information online as it was perceived to be more efficient, which also helps users in making decisions and acting swiftly [27]. Topics on complementary feeding and feeding may also be related to more mothers staying at home and needing to pay higher attention to their older children/infants.

The only form/type that was slightly higher before COVID-19 compared to during COVID-19 was text; meanwhile, there was an increase in video, infographic, and photo posts before COVID-19 compared to during COVID-19; however, these trends were not significant. Consequently, COVID-19 may have no impact on the type or form of breastfeeding information used. In comparison, a study reported that the text-link type was the most popular post type (62.8%), followed by text status (19.8%) and photographs (14.1%) [28]. Even though the link was the most frequently accessed by the user, posts of images and videos produced more user interaction [28].

There appears to be an increase in the total number of posts with the correct information before COVID-19. However, before COVID-19, there was more misleading and mixed information than during COVID-19. During the epidemic, the posts’ tendency for correct information hinted that credible sources could be used. The same can be said for misleading and mixed information, as posts during COVID-19 showed a downward trend. For example, a study on the accuracy of knowledge about gynecologic cancer indicated that 61.7% of the information was true and 38.3% was misinformation before COVID-19 [29]. This is in line with another study where 27.5% of the posts were correct, followed by 22.30%, and, lastly, an unverified 27.50% [13]. Thus, despite the pandemic’s presence, most of the information from trustworthy sources was accurate on social media. However, users need to take extra precautions against misleading information on social media.

Health practitioners, academic institutions, governments, and non-governmental organizations (NGOs) should continue to promote correct and reliable breastfeeding information on social media so that more individuals may seek information from trustworthy sources and dispel breastfeeding myths. In addition, a guideline may be created to teach users how to get and share information online responsibly and ethically. Furthermore, the information and advice given during the face-to-face postpartum health visit can be transformed into an online media version so that information can be shared widely during movement restrictions. For example, an online breastfeeding app could be developed and used by both mothers and healthcare providers to ensure mothers are exposed to reliable sources on the app. This can also provide an opportunity for efficient online follow-up for infants and mothers during the postpartum period.

Study limitations

There are a few limitations to consider. First, this study solely used Facebook as a social media platform; thus, the results do not represent other social media. Further certain data posts did not wholly reflect the breastfeeding content variables; therefore, they must be classified into an existing variable with the same common aspect. Next, the data collection period during COVID-19 was done during the first year of the pandemic; hence, it cannot capture the full impact of the COVID-19 pandemic on online breastfeeding information dissemination during the later years of pandemic. Nevertheless, this study is focusing mostly on the phase when strict movement restrictions or lockdown was implemented, during which online communication was the main medium for mothers to communicate or gather information.

Next, because the data were chosen based on keywords, they may not reflect the whole of breastfeeding information. Even though the data came from Malaysia, this research did not include other ethnic languages than Malay and English. For example, some Facebook groups for breastfeeding moms in the Chinese and Indian communities prefer to communicate in Mandarin or Tamil, limiting the number of Facebook posts included in our research. Furthermore, non-proper or slang terms such as *Bfed*, *myusu*, or *nyusu*, or abbreviated forms such as *dl* (direct latching) cannot be captured to relate to breastfeeding. Hence, some information could be captured by keywords used in this study.

## Conclusions

Sharing personal experiences was the most common post in the context of breastfeeding information, followed by asking questions, advice, and support. There were significant differences in the number of posts regarding sharing personal experiences, asking questions/advice/support, and advertising before and during COVID-19, while the rest remained the same. There was no significant change in the types/forms of breastfeeding information before and during COVID-19, but the engagement of posting is yet unexplored. Nearly half of the data were deemed misleading, while the other half of the data were found to be correct, with a small amount of mixed information in the context of post accuracy. There was a significant change in the post accuracy scale before and during COVID-19. Overall, this study demonstrates that there is considerable breastfeeding information on social media that can be trusted. Nonetheless, public education about the importance of treating internet material with caution remains vital. Future studies should explore the social media context using various social media types such as Instagram and Twitter. In addition, the rate of social media engagement (e.g., likes, comments, and shares) should also be explored to assess the speed of information spread among users. Overall, the present study provides the baseline data for policymakers and scientific communities to plan strategies for improving breastfeeding information. This includes strategizing the most effective way for breastfeeding information dissemination to increase breastfeeding practices. For example, an online breastfeeding app could be developed and used by both mothers and healthcare providers, along with visual guidelines and links to credible web resources and social media networks. This may allow for more efficient online follow-up for infants and mothers during the postpartum period, specifically during a situation of movement restriction such as a disaster or pandemic, as well as provide credible resources for the general public.
